# The Effect of MI Varnish™ on Caries Increment and Dietary Habits among 6- and 12-Year-Old Children in Riga, Latvia: A 3-Year Randomized Controlled Trial

**DOI:** 10.3390/dj10060096

**Published:** 2022-06-01

**Authors:** Jekaterina Gudkina, Bennett T. Amaechi, Stephen H. Abrams, Anda Brinkmane, Eva Petrosina

**Affiliations:** 1 Conservative Dentistry and Oral Health Department, Riga Stradins University, LV-1007 Riga, Latvia; gregory@rms.lv; 2 Department of Comprehensive Dentistry, University of Texas Health Science Center, San Antonio, TX 78229, USA; amaechi@uthscsa.edu; 3 Quantum Dental Technologies and Cliffcrest Dental Office, 2995 Kingston Road Scarborough, Scarborough, ON M1M 1P1, Canada; dr.abrams4cell@sympatico.ca; 4 Statistical Laboratory, Riga Stradins University, LV-1007 Riga, Latvia; eva.petrosina@rsu.lv

**Keywords:** fluoride varnish, caries increment, dietary habits, caries prevalence, oral hygiene

## Abstract

Aims: This randomized controlled trial investigated the effect of MI Varnish™ (5% NaF/CPP-ACP) on caries increment in 6- and 12-year-old children in Riga, Latvia within 36 months. Methods: Forty-eight 6-year-old children (Group 1) and forty-seven 12-year-old children (Group 3) received quarterly varnish application, while forty-eight 6-year-old children (Group 2) and thirty-seven 12-year-old children (Group 4) did not have varnish applied. All children/parents received the same preventive advice. All children were visually examined using ICDAS-II criteria. Questionnaires on dietary habits were completed by the children/parents at baseline and after 36 months. DMFS and dfs were calculated from ICDAS data. The statistical analysis was performed (α = 0.05) using a Chi-squared test, paired *t*-test (Welch test) and the Pearson correlation coefficient. The trial registration number is ISRCTN10584414. Results: In Group 1 versus Group 2, the DMFS(SD) (Baseline/36 months) values were 5.02(5.85)/13.21(6.67) (*p* < 0.001) versus 2.65(4.54)/10.81(6.14) (*p* < 0.001), respectively; the dfs(SD) (Baseline/36 months) values were 36.75(12.96)/24.04(12.9) (*p* < 0.001) versus 33.67(12.74)/23.88(11.91) (*p* < 0.001), respectively. In Group 3 versus Group 4, the DMFS(SD) (Baseline/36 months) values were 48.62(23.18)/70.96(23.28) (*p* < 0.001) versus 34.73(17.99)/54.95(16.09) (*p* < 0.001), respectively; the dfs(SD) (Baseline/36 months) values were 1.7(4.4)/0 (*p* < 0.05) versus 2(6.39)/0 (*p* = 0.06), respectively. The prevalence of caries (dfs + DMFS) decreased by 4.52 (*p* < 0.001) and 1.63 (*p* < 0.001) in Groups 1 and 2, respectively, but increased by 20.64 (*p* < 0.001) and 18.22 (*p* < 0.001) in Groups 3 and 4, respectively. An analysis of the questionnaires indicated the habitual, frequent consumption of a sugary diet by all the children. A significant correlation (*r* = 0.321; *p* < 0.05) was observed between caries increment and the frequency of daily intake of sugary snacks, soft drinks and tea with sugar at baseline only in Group 1. Conclusions: A quarterly application of MI varnish (CPP-ACP/fluoride) reduced caries increment in 6- and 12-year-old children in Riga, Latvia.

## 1. Introduction

The relationship between the intake of dietary sugar and oral health has been of scientific concern since the early 1900s [1]. Fermentable carbohydrates have recently been described as the most relevant common dietary risk factor for both periodontal disease and dental caries [1]. The popularity of tea drinking, usually with sugar, in Latvia is considered historical and cultural, and it is also related to the cold climate in both summer and winter. Almost 80–90% of the 6- and 12-year-old children drink tea. A majority (approximately 70–80%) of 6- and 12-year-olds use a mean of 4.7 and 7.3 teaspoons of sugar daily, respectively [2]. Just as the consumption patterns of soft drinks have demonstrated an increase in many countries, so this is also the situation in Latvia [2]. A recent study found a high prevalence and severity of caries among 12-year-old children in Latvia, and this prevalence was higher than that of their European counterparts [3]. Therefore, there is a need to implement evidence-based interventions to decrease the prevalence and severity of dental caries in Latvian children. Unfortunately, there are no preventive programs in Latvia or Riga, its capital, not even community-water fluoridation [4]. The government supports only one visit to a hygienist per year for each child. However, children between the ages of 7 and 12 years may be allowed two visits per year. Nevertheless, all hygiene procedures are sponsored only if performed by the hygienist and not the dentist, so all preventive procedures rest on the few available hygienists [4]. For this reason, the major preventive strategy remains personal dietary control and oral hygiene procedures, which suffer limited noncompliance, especially dietary control. For this reason, a professional preventive program is required to combat the high prevalence of caries among children in Latvia [4].

Fluoride varnish (FV) is considered safe, well-accepted by children, and easily delivered by health practitioners [5,6]. Usually, fluoride varnish applications are targeted at children at high caries risk, and it is currently considered complementary to other forms of fluoride interventions, such as fluoridated toothpaste and fluoridated water [6,7]. A therapeutic product combining fluoride and CPP-ACP in a varnish (MI Varnish™, GC corporation, Tokyo, Japan) was developed a few years ago. Previous studies have demonstrated its caries prevention potential [8].

The aim of the present study was to investigate the effect of MI Varnish™ on caries prevention in 6- and 12-year-old children in Riga, Latvia within 36 months. We hypothesized that a quarterly application of MI Varnish™ could reduce caries increment in Latvian children in Riga.

## 2. Materials and Methods

### 2.1. Trial Design

This randomized controlled parallel study was conducted between February 2016 and March 2020, in both age groups, with the allocation ratio of 1:1—varnish group to control group.

### 2.2. Participants

The study was performed at the RSU Institute of Stomatology in Riga, on a population of 6- and 12-year-old inhabitants of Riga who visited the Institute for dental treatment. RSU Ethics Committee approval (#22/17 December 2015) was obtained. Written informed consent and assent were obtained from parent and child, respectively.

### 2.3. Randomization

All children were recruited into the study in a random manner during their regular dental check-ups, as follows. During the visit, the children were listed by serial numbers in the order of their arrival at the reception desk. On meeting the examiner, the odd numbers of both age groups were recruited into the Varnish group (6 years old into Group 1; 12 years old into Group 3), while the even numbers were recruited into the control groups (6 years old into Group 2; 12 years old into Group 4). The information about this study was delivered to the children and their parents at the time of recruitment. The children’s demographic information was collected, including their full name with the first two letters of their surname, an available mobile telephone number, and an e-mail address, and the information was filed in a specially prepared study folder for each subject. Complete names and surnames were only provided in the signed, written informed consent before the clinical examination and procedure. The data on the demographic and clinical characteristics are provided in Table 1.

### 2.4. Inclusion and Exclusion Criteria

Every child within the chosen ages who visited the Institute was eligible to be enrolled irrespective of their caries status, except when the child and/or the parent declined to participate in the study at any point, the families moved away from Riga, or the children or parents did not answer the three telephone calls confirming their appointments. Also excluded were children wearing orthodontic braces or diagnosed with general ill health within the study period. 

### 2.5. Recruitment

Recruitment was facilitated by exempting volunteers from the two-year wait time required for their regular, complete (including radiographic examination) dental check-up financially supported by the government. 

### 2.6. Sample Size Calculations

To calculate the sample size, we estimated the margin of error at 5% at 95% confident interval. The standard deviation was estimated at 5 from previous studies [4]. Since variables are quantitative continuous independent data between two groups, the following formula was used for calculating the sample size for comparing two groups [9,10]: the standard deviation from previous studies was 5; Zα = 1.96 (from Z table at a selected level of significance of 5%); Zβ = 0.842 (from Z table at 80% power); effect size (ES) = 3, the difference between mean values [9,10]. The level of significance was selected at 5% and the power of study at 80%, and the suitable statistical test in this condition was a two-tailed unpaired *t*-test [9,10]. The estimated sample size was 64 children per group. Dropout was calculated at 25%. The lowest power of study was achieved obtaining the primary endpoints (DMFS, dfs, DMFS + dfs using ICDAS II criteria) and exceeded 80%.

### 2.7. Interventions

Prior to the clinical examination, a benchmark examiner who is an experienced Cariologist (expert in caries management) used the first 15 patients, not included in the study, to calibrate the clinical examiner (JG) on visual caries examination. Intra-examiner agreement was determined using the examiner’s repeated examination of 10 of the 15 of patients over a period of time and was determined by comparing the caries scores between the examiner and the benchmark examiner (the trainer). Agreement to the set standard was quantified by Kappa analysis [11], with intra-examiner and inter-examiner (trainer–trainee) scores of 0.81 and 0.87, respectively (any score > 0.70 was considered to be acceptable as adequate agreement).

At baseline, all subjects were visually examined by the calibrated dentist (JG). The examination environment, procedure, and sequence employed during normal dental check-up were maintained throughout the study, including protocols for infection control and sterilization. A tooth was deemed to be present in the oral cavity when part of its occlusal surface was visible without the need for gingival displacement. A visual caries assessment was performed on every surface of each tooth in all subjects. Detected lesions were recorded in a specially designed case report form (CRF).

The examiner used the caries assessment criteria of the ICDAS II [12]: score 0: sound tooth surface; score 1: first visual change (opacity or discoloration) in enamel, hardly visible on the wet surface but distinctly visible after air drying; score 2: distinct visual change (opacity or discoloration) in enamel, visible without air drying; score 3: localized enamel breakdown without visible dentin; score 4: underlying dark shadow from dentin without cavitation; score 5: distinct cavity with visible dentin; score 6: extensive distinct cavity with visible dentin. The same scoring criteria were used for caries around restorations.

Prior to examination, the subjects brushed their teeth with non-fluoridated professional toothpaste (Zircate Prophy Paste, Dentsply Caulk, Konstanz, Germany). Following brushing, an examination was carried out on clean, plaque-free teeth with 5 s drying of each tooth surface to identify early lesions. Each examined surface was placed under one of the following classifications: sound, non-cavitated (n/c) lesion (ICDAS 1 and 2), non-cavitated lesion around restorations (CARn/c), cavitated (c) lesion (ICDAS II 3–6), or cavitated lesion around restorations (CARc). It is worth mentioning that surfaces of teeth that were unerupted at baseline were not included in further statistical analysis.

Following visual examination, digital bitewing (BW) radiographs were provided to every child and were examined at the Department of Radiology at the RSU Institute. The number of BW radiographs were determined by the eruption of second permanent molars. For this reason, the 6-year-olds only had two BW radiographs, while the 12-year-olds had four BW radiographs with the eruption of their second permanent molars. At the end of the examination procedure, the BW radiographs were analyzed and explained to the participants. All data collected with regard to caries and oral hygiene status were placed in the official dental patient chart.

### 2.8. Blinding

All examinations, as well as MI Varnish application procedures, were provided by one and the same Clinical Examiner in all groups at baseline and at the 36-month examination. The Clinical Examiner and the Recorder were blinded at baseline and at the 36-month visits with regard to each child’s study group (MI Varnish or Control groups). Neither the children nor their parents in the Varnish groups (Groups 1 and 3) were informed about the name of the varnish (MI Varnish) used or the manufacturer’s name.

### 2.9. Study Treatment

Following the baseline caries examination, the treatment groups (Groups 1 and 3) received an application of MI Varnish™ (5% sodium fluoride, GC Corp., Tokyo, Japan), while the control groups (Groups 2 and 4) did not have varnish applied. The application of the MI Varnish was performed in accordance with the manufacturer’s instruction. The manufacturer’s post-varnish instruction was given to all children and their parents. 

Subsequently, subjects in the treatment groups (Groups 1 and 3) were recalled every 3 months for re-application of the varnish. However, all children and their parents, irrespective of their groups, received the same general preventive advice at baseline and at the 36-month examination.

Children and their parents were informed about the precise time of their subsequent visits by telephone call using the mobile telephone number provided at baseline. At the baseline and three monthly MI Varnish re-application visits, teeth were brushed with non-fluoridated professional toothpaste (Zircate Prophy Paste; Dentsply Caulk, Konstanz, Germany), and MI Varnish™ (GC Corp., Tokyo, Japan) was re-applied on all tooth surfaces.

### 2.10. Assessment of Oral Hygiene

The Greene–Vermillion oral hygiene index (G-V index), as described by the World Health Organization [13], was used to determine the oral hygiene level in all participants at baseline and at the 36-month visits. All subjects received general oral hygiene instruction at baseline and at the last visit. Due to the incomplete eruption of the first molars in the 6-year-old population, it was only possible to measure the G-V index in 31.3% (*n* = 15) of Group 1 participants at baseline and 93.75% (*n* = 45) at the 36-month visit. For the same reason, it was only measured in 16.67% (*n* = 8) of Group 2 participants at baseline and 97.92% (*n* = 47) at the 36-month visit. In 12-year-olds, due to early extraction and, hence, the loss of the first molars, the G-V index was measured in 89.36% (*n* = 42) of participants at baseline and at 36 months in Group 3. However, in Group 4 at baseline and at the 36-month visit, the G-V index was measured in all participants.

### 2.11. Assessment of Dietary Habits

**Figure 1:**
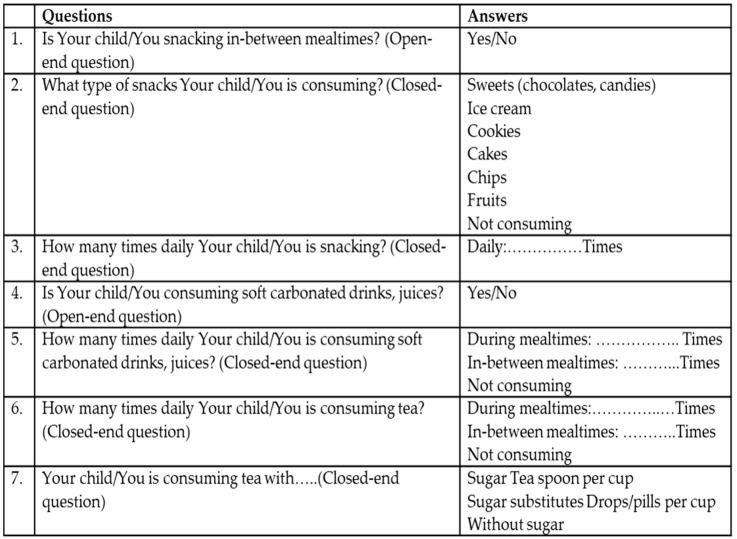
Questionnaire of dietary habits in 6- and 12-year-olds.

A questionnaire was used to obtain information on dietary habits. The questionnaire was administered to all participants at baseline and at the 36-month visits. Depending on age, the children and/or their parents were questioned about snacking habits, intake of chocolates, consumption of carbonated soft or sport drinks during the day, number of teaspoons of sugar (t.s.) per cup of tea, and the number of cups of tea consumed daily. The questionnaire consisted of a variety of open-ended and closed-ended questions (Figure 1). The questionnaire was administered while the children were waiting with their parents to take radiographs, so although all questions about oral hygiene and dietary habits were answered by the children in both age groups, their parents may have helped with the responses. In 12-year-old children, all questions about dietary habits were answered by the children, although their parents may have helped with the responses. Parents provided all responses for the 6-year-old children. No information about the so-called “Teeth Healthy Diet” (https://www.acffglobal.org/projects/dietary-counselling accessed on 1 February 2019) was provided at baseline or throughout the study period. However, the advice to improve on teeth-healthy dietary habits was provided only at the last visit, after the questionnaire had been filled out and the oral examination was completed.

### 2.12. Statistical Methods

For the statistical analysis, the ICDAS-II data were used to calculate the DMFS/dmfs (D = decayed surfaces, M = Missing surfaces, and F = Filled surfaces), with Decayed (D) being n/c + c + CARn/c + CARc ((non-cavitated (n/c) lesion (ICDAS 1 and 2), non-cavitated lesion around restorations (CARn/c), cavitated (c) lesion (ICDAS II 3–6), and cavitated lesion around restorations (CARc)). Data were analyzed using SPSS software package IBM SPSS Statistics v.22, R Studio 2021.09.1, and Excel 2013; *p*-values less than 0.05 were considered statistically significant. For the primary outcomes, mean values of parameters concerning dental caries development and oral hygiene were obtained using a *t*-test at baseline and at the 36-month period in all groups. The difference in dietary habits at baseline and at the 36-month period was calculated using a Chi-squared test in all groups. The statistically significant difference between mean parameters in a period of three years was obtained using a *t*-test for paired parameters (Welch test) (α = 0.05) in all groups. For secondary outcomes, a Pearson correlation coefficient (α = 0.05) analysis was used to determine any statistically significant difference between sugar consumption and caries development at baseline and at the 3-year period in all groups. The trial registration number is ISRCTN10584414 (https://www.isrctn.com/ISRCTN10584414 (7 March 2019)).

## 3. Results

### 3.1. Flow Diagram and the Number of Analyzed Participants

**Figure 2:**
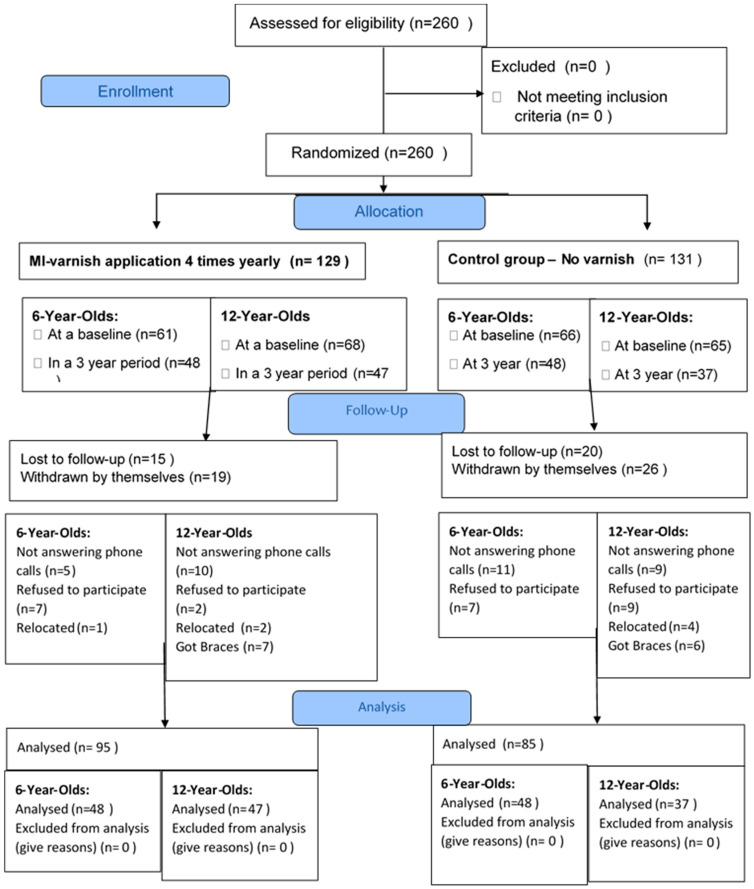
Participant flow diagram for 6- and 12-year-olds.

A flow diagram of the study is shown in Figure 2. In 6-year-old children, the dropout was 21.31% (*n* = 13) in the Varnish group and 27.3% (*n* = 18) in the Control group. In 12-year-old children, the dropout was 30.88% (*n* = 21) in the Varnish group and 43.07% (*n* = 28) in the Control group. The following numbers of subjects completed the study in each group: Group 1 (48); Group 2 (48); Group 3 (47), and Group 4 (37), and their data were used for the statistical analysis.

### 3.2. Outcomes

#### 3.2.1. Primary Outcomes

As stated above, the ICDAS-II data were used to calculate the DMFS and dfs for each group with a 95% Confidence Interval (95% CI) (Table 2 and Table 3). 

**Figure 3:**
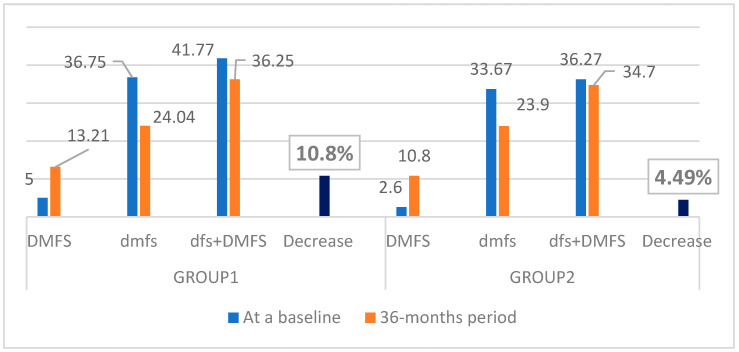
Changes in caries increment in a period of 3 years in 6-year-olds (Group 1 and Group 2).

**Figure 4:**
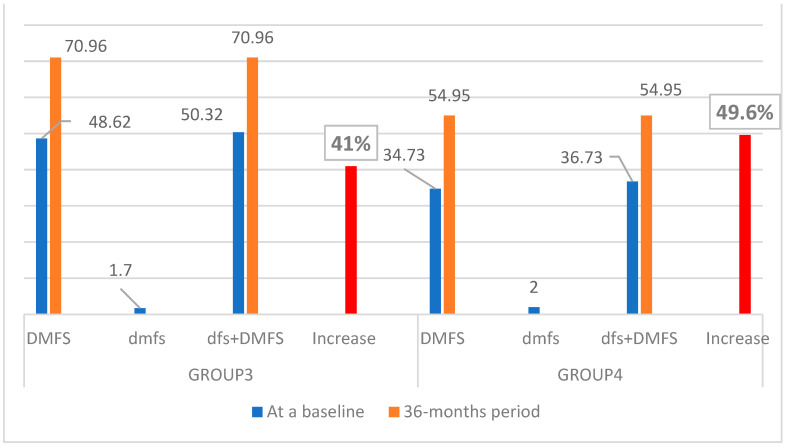
Changes in caries increment in a period of 3 years in 12-year-olds (Group 3 and Group 4).

In 6-year-olds, the mean DMFS increased 2.6 times in Group 1 and 4.1 times in Group 2, while the mean dfs decreased 0.65 times and 0.7 times in Group 1 and Group 2, respectively. In 12-year-olds, the mean DMFS increased 1.46 times in Group 3 and 1.6 times in Group 4, while the dfs decreased 1.7 times and 2.0 times in Group 3 and Group 4, respectively. Based on the DMFS and dfs, the overall prevalence of caries decreased by 10.8% (4.52) in Group 1 and 4.49% (1.63) in Group 2 (Figure 3) and increased by 41% in Group 3 and 49.6% in Group 4 (Figure 4). Generally, a decrease in caries increment was obvious in primary dentition in all groups (Figure 3 and Figure 4). However, in permanent dentition, caries increment increased at a slower rate in Group 1 and Group 3 (undergoing MI Varnish treatment) compared to Group 2 and Group 4 (Figure 3 and Figure 4).

With regard to the questionnaire on dietary habits, Table 2 and Table 3 further show the results of the data collected on the number of teaspoons of sugar per cup of tea, while Table 4 and Table 5 show the results of the analysis of the dietary data from Groups 1, 2, 3, and 4 at baseline and at the 36-month visits. The questionnaire analysis indicated the habitual, frequent consumption of a sugary diet by the children (Table 4 and Table 5). 

#### 3.2.2. Secondary Outcomes

Table 6 presents the results of the correlation analysis between sugar consumption and caries development in all four groups at baseline and at the 36-month period. The analysis indicated a significant correlation (*r* = 0.321; *p* < 0.05) between caries increment and the frequency of daily intake of sugary snacks, soft drinks and tea with sugar at baseline only in Group 1. Caries development parameters were divided into Caries 1 (n/c + c + CARn/c + CAR/c) and Caries 2 (n/c + c + CARn/c + CAR/c + filled(F + f)). Parameters of sugar consumption included in this correlation were number of teaspoons of sugar per day (“Sugar daily amount”) and daily intake of snacks and soft drinks and consumption of tea with sugar (“Sugar frequency”). 

### 3.3. Adverse Effects

No evidence of any adverse effect was detected or reported with the quarterly application of MI Varnish™ (5% sodium fluoride, GC Corp., Tokyo, Japan).

## 4. Discussion

Biofilm removal and good dietary habits play a significant role in caries control [4]. In the present study, in a period of 3 years, the moderate level of oral hygiene remained unimproved in 6-year-olds (Table 2). The absence of statistical significance could be explained by the reduced number of 6-year-olds with an estimated Greene–Vermillion oral hygiene index at baseline due to incomplete eruption of some permanent teeth (central permanent incisors and first permanent molars).

In 12-year-olds, a slight, statistically significant improvement of oral hygiene level (from 1.62 (0.52) to 1.22 (0.62)) was observed in Group 3 participants, who were offered quarterly MI Varnish applications that came with professional cleanings (Table 4). The surprising improvement in oral hygiene in Group 4 (from 1.56 (0.49) to 1.38 (0.54)) may be attributed to the enlargement of the sample size. However, the results in Group 3 are promising and suggest that regular visits to the dentist/hygienist with regular fluoride treatment may influence improvements in the level of oral hygiene with its consequent decrease in caries increment. However, it needs to be emphasized that this study was performed in the afternoon, after school, when the children had already eaten their lunch and snack and had consumed soft drinks and sugared tea during the school hours, and clearly, not all the children changed their dietary habits or brushed their teeth during this period. Moreover, in two previous studies conducted in Riga, a similar Greene–Vermillion index was used to determine the level of oral hygiene, and it only reached a moderate level at baseline [2,4], with no improvements in a 3-year period [4].

Dietary habits have shifted in all age groups in Western populations in recent decades, including a nearly doubled intake of energy-dense, low-nutrient snack foods [14,15]. In children, more than 30% of daily energy intake was reported to come from such foods, and on average, 75% of Americans report daily snacking [16]. In our study, changes in dietary habits did not achieve statistically significant results within a period of 3 years in any of the groups (Table 3 and Table 4). Moreover, in a period of 3 years, all the children showed a slight reduction in sugary snack consumption, but at the same time, the non-snacking habit increased sufficiently only in the Control group of 12-year-olds (Table 4 and Table 5). There was no reduction observed in the consumption of soft drinks in any of the groups within the period of this study (Table 4 and Table 5). No significant changes were observed in tea-drinking habits, number of cups of tea consumed, number of teaspoons of sugar per cup, or daily amount of sugar consumed in any of the groups within the 3-year period (Table 2 and Table 3). At the same time, only the intake of fruits showed a slight improvement in the dietary habits of all groups (Table 4 and Table 5). However, children in both age groups who received MI Varnish showed a slight reduction in the frequency of consuming sugary snacks, soft drinks, and tea with sugar within the 3-year period, but again, with no statistical significance (Table 4 and Table 5).

It is pertinent to mention that only these types of sugar consumption parameters were possible to calculate in all examined children at baseline and at the 36-month period. No information was provided on the name/manufacturer of snacks and soft drinks consumed, the number of snacks consumed, or the amount of soft drink consumed during one snacking occasion. This fact could be counted as one of our trials’ limitations. Moreover, comparing our findings to the study conducted in 2006–2008 in Riga [2], it was obvious that the frequent consumption of sugary snacks and carbonated soft drinks, as well as drinking tea with sugar, remained at a high level and needs to be reduced. Unfortunately, no efforts were made in educating children about teeth-healthy diets within previous years, from 2008 [2] to 2020. Both the amount and frequency of sugar intake are risk factors for the development of dental caries [17]. Advice to limit the frequency of intake of free sugars is, however, an important part of patient dental health education at the level of the individual [17,18]. Moreover, in our study, we advised every child, only at the last visit of the study, to maintain the so-called ”teeth healthy diet” (https://www.acffglobal.org/projects/dietary-counselling. accessed on 1 February 2019)

As a secondary outcome in our study, the correlation between dental caries parameters and “Sugar-intake frequency” showed a statistically significant relationship, but only at baseline and only in 6-year-olds who received MI Varnish (Table 6). However, this established relationship disappeared over the 3-year period in 6-year-olds who received MI Varnish due to the protection provided by the regular application of MI Varnish during this study period. The lack of statistically significant results at baseline in 6-year-olds in the Control group and both groups of 12-year-olds could be explained by the prevalence of caries that differs from 27.73% (12-years-olds in the Control group) to 35.42% (12-year-olds who received MI Varnish), and to 36% (6-year-olds in the Control group), but it is 38% in 6-year-olds who received MI Varnish. Moreover, in a 3-year period, the prevalence of caries differs from 30% (6-year-olds in the Control group) to 39% (12-year-olds in the Control group).

It could be suggested that the higher the prevalence of caries, the stronger the relationship with the frequency of sugary snacks and drinks consumption (soft drinks and tea with sugar), and caries parameters could be established. At the same time, 12-year-olds in the Control group showed results very close to statistical significance in the correlation analysis at baseline, as well as at the 3-year period (Table 6), and this may mean that the enlargement of the study sample of 12-year-olds in the Control group would have shown a statistically significant linkage between dental caries parameters and “Sugar-intake frequency” (Table 6).

In our study, only “Sugar-intake frequency” as a sugar consumption parameter showed statistical significance compared to “Sugar daily amount”, which was also used as another sugar consumption parameter (Table 6). Notwithstanding that a statistically significant correlation was observed between dental caries parameters and “Sugar-intake frequency”, it could be suggested that dietary questionnaires in future trials should contain more detailed information about the consumption of sugar in its pure form and sugar-containing products that were used daily on a regular basis. This fact could be counted as one of our trials’ limitations. 

A crucial step in the development of a caries care plan involving non-operative care of lesions is caries diagnosis, distinguishing the cavitated and non-cavitated lesions [19]. In our previous studies, the Radke method [2,4] and ICDAS II [4] were used as caries assessment methods in 6- and 12-year-old age groups. Notwithstanding that the Radke method was used to assign dental caries at baseline and in a 3-year period, caries experience (DMFS-Radke criteria) has doubled in 6- and 12-year-old children in Riga [4]. Therefore, it could be concluded that children at the ages of 6 and 12 are at high caries risk in Riga [2,4].

In our study, in 12-year-olds, the increase in the “Decayed” surfaces parameter (D) of permanent teeth was obvious in both groups: 1.4 times in Group 3 and 1.6 times in Group 4 (Table 3; Figure 4). The difference between both groups seems to be a small value, but it could be explained by the difference in sample size; there were only 37 children in the Control group. In 6-year-olds in a 3-year period, caries increase and decrease were observed in permanent and primary teeth, respectively. The decrease in the “decayed” surfaces parameter in primary teeth is clearly visible in both groups at the age of 6, and it could be explained by exfoliation of the majority of primary teeth within this study period (Table 2, Figure 3). If long-term results (3-year period) of the preventive effect of MI Varnish in primary dentition need to be studied, then a baseline examination should be performed in the early age population (e.g., 3 years of age), when the primary dentition has been completely established and remains unchanged until the age of 6. However, the question then arises about the anxiety of early-aged children in the dental chair. At the same time, looking at the “Decayed” surfaces parameter in permanent teeth in 6-year-olds in both groups, it increased 2.5 times and 4.5 times in the children who received MI Varnish and in the Control group, respectively (Table 2; Figure 3). The gained results could be explained by the long-term and regular application of MI Varnish.

A Cochrane systematic review, analyzing only 22 clinical trials due to the inclusion criteria, has shown that fluoride-containing varnish provides caries protection of 43% in young permanent teeth and 37% in primary teeth [20] with F-varnish application from two to four times yearly [18]. This relative effect has been noticed in populations with different levels of caries risk and exposure to other sources of fluoride [20]. The evidence produced showed moderate quality due to issues with the trial designs [20]. Notwithstanding that our study showed a rather small percentage of caries protection in Groups 1 and 3 compared to the Cochrane review, this fact could be explained by the different size of the sample (9595 children were analyzed), age groups (up to 16 years of age), and longevity (at least one-year results) [20], and by the number of limitations of our study—a small sample size and a large dropout in Group 4.

Fluoride treatment is still the standard therapy for remineralization of caries lesions [6,21,22]. MI Varnish is a 5% NaF varnish containing CPP–ACP, with calcium and phosphate being essential minerals for remineralization, while fluoride plays a crucial role in enhancing the process [6]. Fluoride has been added to the CCP-ACP formulation to increase its remineralization efficacy [6,23] compared with a fluoride treatment alone [24]. New rebuilt crystalline structures composed of fluoridated hydroxyapatite and fluorapatite are characterized by a higher resistance to acid attack than the original ones [24,25] and can be successfully used in the remineralization of early carious lesions [26,27], as well as in reducing dentin hypersensitivity [28,29].

At the end, the quarterly application of fluoride varnish resulted in a decreased rate of caries progression in permanent dentition in Group 1 and Group 3 (Figure 2 and Figure 3). For this reason, it could be seen as a “first step” in the preparation of a preventive program in Riga and then in Latvia. The observed positive preventive results in both age groups that received MI Varnish could be attributed to the regular visits (every 3 months) to the dental office for MI Varnish application and the removal of dental plaque, as well as the change in attitude of the children and their parents toward dental health from negative (high caries rate) to positive.

All children were recruited into the study in a random manner during their regular dental check-ups. Every child within the chosen ages who visited the RSU Institute of Stomatology was eligible to be enrolled irrespective of their caries status. In other words, the findings of this study could be applied to a broad context. We intend to use the obtained results to prepare a preventive program in Riga in the future. The application of MI Varnish was performed in children at a high caries risk, with no changes in their dietary and oral hygiene habits and skills. Therefore, the obtained results of our study concerning caries increment in children of both age groups undergoing MI Varnish treatment, as it was hypothesized, could be counted as a strength of our study. However, on the other hand, the question of its cost-effectiveness arises, and it should be reconsidered and well-discussed before implementing it in the population/society as a Public Health measure.

In conclusion, the present study demonstrated that the quarterly application of varnish containing CPP-ACP and fluoride reduced caries increment, despite challenging dietary habits, among 6- and 12-year-old children in Riga, Latvia. It is anticipated that the result of this study will prompt the government to develop a caries preventive program based on topical fluoride application, in particular, fluoride varnish.

## Figures and Tables

**Table 1:** Demographic and clinical characteristics for each group at baseline.

	Group 1 (*n* = 61)	Group 2 (*n* = 66)	Group 3 (*n* = 68)	Group 4 (*n* = 65)
Male	44.3% (*n* = 27)	53% (*n* = 35)	55.9% (*n* = 38)	46.2% (*n* = 30)
Female	55.7% (*n* = 34)	47% (*n* = 31)	44.1% (*n* = 30)	53.8% (*n* = 35)

**Table 2:** Mean values (SD) of ICDAS II data, Greene–Vermillion index (G-V ind.), and teaspoons of sugar used per cup and daily at baseline and at 36 months among the 6-year-old children (Group 1 and Group 2).

	Group 1 (*n* = 48) (MI Varnish)			Group 2 (*n* = 48) (Control)		
	At Baseline	At 36 Months	*p* Values	At Baseline	At 36 Months	*p* Values
n/c	27.6(9.4) 95% CI [24.4, 29.6]	28(10.28) 95% CI [25.2, 30.8]	*p* = 0.813	26.1 (7.26) 95% CI [24,28]	27.02(9.4) 95% CI [24.4, 29.6]	*p* = 0.59
c	6.58 (7.16) 95% CI [4.02, 7.98]	3.15 (3.18) 95% CI [2.15, 3.85]	*p* < 0.001	5.81 (7.73) 95% CI [3.02, 6.98]	2.23 (2.5) 95% CI [1.43, 2.57]	*p* = 0.001
f	7.13 (5.91) 95% CI [5.59, 8.41]	6.38 (4.7) 95% CI [4.87, 7.13]	*p* = 0.39	4.46 (4.63) 95% CI [2.87, 5.13]	5.08 (4.5) 95% CI [3.87, 6.13]	*p* = 0.427
CARn/c	0.08 (0.28) 95% CI [0, 0]	0.04 (0.2) 95% CI [0, 0]	*p* = 0.322	0.21 (0.5) 95% CI [0, 0]	0.15 (0.41) 95% CI [0, 0]	*p* = 0.444
CAR/c	0.98(1.33) 95% CI [0.717, 1.28]	0.1 (0.37) 95% CI [0, 0]	*p* < 0.001	0.85 (1.77) 95% CI [0.717, 1.28]	0.19 (0.53) 95% CI [0, 0]	*p* = 0.012
DMFS	5.02 (5.85) 95% CI [3.59, 6.41]	13.21 (6.67) 95% CI [11.3, 14.7]	*p* < 0.001	2.65 (4.54) 95% CI [0.87, 3.13]	10.81 (6.14) 95% CI [8.3, 11.7]	*p* < 0.001
Decayed	4.81 (5.55) 95% CI [2.59, 5.41]	12.17 (5.87) 95% CI [10.6, 13.4]	*p* < 0.001	2.27 (4.1) 95% CI [0.87, 3.13]	9.44 (5.8) 95% CI [7.59, 10.4]	*p* < 0.001
F	0.54 (2.38) 95% CI [0.434, 1.57]	1.02 (1.91) 95% CI [0.717, 1.28]	*p* = 0.23	0.06 (0.32) 95% CI [0, 0]	0.92 (1.69) 95% CI [0.717, 1.28]	*p* = 0.001
M	(0.00)	0.00(0.00)	*p*-konst.	0 (0)	0.42 (2.89) 95% CI [0, 0.566]	*p* = 0.32
dfs	36.75 (12.96) 95% CI [32.6, 39.4]	24.04 (12.9) 95% CI [20.6, 27.4]	*p* = 0.214	33.67 (12.74) 95% CI [29.6, 36.4]	23.88 (11.91) 95% CI [19.9, 26.1]	*p* = 0.002
d	28.81 (12.98) 95% CI [25.1, 32.5]	17.56 (10.39) 95% CI [14.6, 20.5]	*p* < 0.001	28.27 (10.41) 95% CI [25.3, 31.2]	19.65 (10.09) 95% CI [16.8, 22.5]	*p* < 0.001
f	6.81 (5.64) 95% CI [5.21, 8.41]	5.44 (4.82) 95% CI [4.08, 6.8]	*p* = 0.147	5.38 (6.48) 95% CI [3.55, 7.21]	4.23 (3.94) 95% CI [3.12, 5.34]	*p* = 0.241
All examined surfaces	88.81 (8.78) 95% CI [86.3, 91.3]	93.38 (10.82) 95% CI [90.3, 96.4]	*p* = 0.027	85.1 (11.56) 95% CI [81.8, 88.4]	96.98 (10.98) 95% CI [93.9, 100]	*p* < 0.001
G-V index	1.42 (0.47) 95% CI [1.29, 1.55]	1.48 (0.47) 95% CI [1.35, 1.61]	*p* = 0.259	1.65 (0.46) 95% CI [1.52, 1.78]	1.51 (0.41) 95% CI [1.39, 1.63]	*p* = 0.578
Teaspoons of sugar per cup of tea	1 (0.73) 95% CI [0.793, 1.21]	1 (0.82) 95% CI [0.768, 1.23]	*p* = 0.346	0.79 (0.76) 95% CI [0.785, 1.22]	0.88 (0.9) 95% CI [0.717, 1.28]	*p* = 0.615
Daily number of teaspoons of sugar (“Sugar daily amount”)	2 (2.7) 95% CI [1.43, 2.57]	1.68 (1.7) 95% CI [1.43, 2.57]	*p* = 0.300	1.62 (1.95) 95% CI [1.43, 2.57]	1.66 (1.9) 95% CI [1.43, 2.57]	*p* = 0.914
Daily number of cups	1.38 (1.38) 95% CI [0.434, 1.57]	1.11 (1.08) 95% CI [0.717, 1.28]	*p* = 0.143	1.24 (1.25) 95% CI [0.717, 1.28]	1.04 (1.1) 95% CI [0.717, 1.28]	*p* = 0.323

**Table 3:** Mean values (SD) of ICDAS II data, Greene–Vermillion index (G-V ind.), and teaspoons of sugar used per cup and daily at baseline and at 36-months among the 12-year-old children (Group 3 and Group 4).

	Group 3 (*n* = 47)	(MI Varnish)		Group 4 (*n* = 37)	(Control)	
	At Baseline	At 36 months	*p* Values	At Baseline	At 36 Months	*p* Values
n/c	38.26 (13.81) 95% CI [34.3, 42.2]	55.09 (13.86) 95% CI [51.1, 59.1]	*p* < 0.001	30.73 (14.53) 95% CI [26.1, 35.4]	46.73 (11.28) 95% CI [43.1, 50.4]	*p* < 0.001
c	3.64 (4.91) 95% CI [2.24, 5.04]	2.77 (2.86) 95% CI [1.95, 3.59]	*p* = 0.177	1.95 (2.32) 95% CI [1.2, 2.7]	2.22 (4.02) 95% CI [0.92, 3.52]	*p* = 0.643
f	6.38 (7.47) 95% CI [4.24, 8.52]	10.45 (14.49) 95% CI [6.31, 14.6]	*p* = 0.001	3.7 (3.66) 95% CI [2.52, 4.88]	5.49 (4.81) 95% CI [3.94, 7.04]	*p* = 0.003
CARn/c	0.57 (1.23) 95% CI [0.218, 0.922]	0.81(1.44) 95% CI [0.398, 1.22]	*p* = 0.11	0.32 (0.63) 95% CI [0.117, 0.523]	0.32 (0.71) 95% CI [0.091, 0.549]	*p* = 1.000
CAR/c	0.34 (0.84) 95% CI [0.1, 0.58]	0.21 (0.81) 95% CI [0.0, 0.442]	*p* = 0.429	0.16 (0.55) 95% CI [0.00, 0.337]	0.054 (0.23) 95% CI [0.00, 0.128]	*p* = 0.291
DMFS	48.62 (23.18) 95% CI [42, 55.3]	70.96 (23.28) 95% CI [64.3, 77.6]	*p* < 0.001	34.73 (17.99) 95% CI [28.9, 40.5]	54.95 (16.09) 95% CI [49.8, 60.1]	*p* < 0.001
Decayed	41.3 (17.19) 95% CI [36.4, 46.2]	58.89 (14.9) 95% CI [54.6, 63.1]	*p* < 0.001	31.49(16.68) 95% CI [26.1, 36.9]	49.32 (13.71) 95% CI [44.9, 53.7]	*p* < 0.001
F	5.92 (7.13) 95% CI [3.88, 7.96]	10.45 (14.49) 95% CI [6.31, 14.6]	*p* = 0.001	3.24 (3.57) 95% CI [2.09, 4.39]	5.49 (4.81) 95% CI [3.94, 7.04]	*p* < 0.001
M	1.32 (3.29) 95% CI [0.379, 2.26]	1.62 (4.38) 95% CI [0.37, 2.87]	*p* = 0.177	0 (0)	0.14 (0.82) 95% CI [0.0, 0.404]	*p* = 0.324
dfs	1.7 (4.4) 95% CI [0.44, 2.96]	0 (0)	*p* = 0.011	2 (6.39) 95% CI [0.07, 3.93]	0 (0)	*p* = 0.065
d	1.45(3.91) 95% CI [0.592, 2.31]	0 (0)	*p* = 0.015	1.51(5.3) 95% CI [0.0, 3.22]	0 (0)	*p* = 0.091
f	0.23 (0.76) 95% CI [0.0, 0.217]	0 (0)	*p* = 0.040	0.49 (1.39) 95% CI [0.042, 0.938]	0 (0)	*p* = 0.040
All examined surfaces	116.6 (12.61) 95% CI [113, 120]	124.92 (7.68) 95% CI [123, 127]	*p* < 0.001	113.54 (12.54) 95% CI [110, 118]	126.19 (4.39) 95% CI [125, 128]	*p* < 0.001
G-V index	1.62 (0.52) 95% CI [1.62, 1.62]	1.22 (0.62) 95% CI [1.22, 1.22]	*p* < 0.001	1.56 (0.49) 95% CI [1.56, 1.56]	1.38 (0.54) 95% CI [1.38, 1.38]	*p* = 0.046
Teaspoons of sugar per cup of tea	1.31 (0.88) 95% CI [1.31, 1.31]	1.16 (0.89) 95% CI [1.16, 1.16]	*p* = 0.371	1.36 (0.95) 95% CI [1.36, 1.36]	1.17 (1.1) 95% CI [0.848, 1.49]	*p* = 0.51
Daily number of teaspoons of sugar (“Sugar daily amount”)	2.59 (2.5) 95% CI [2.02, 3.16]	2.16 (2.17) 95% CI [1.59, 2.73]	*p* = 0.296	2.67 (2.39) 95% CI [2.03, 3.31]	2.86 (3.3) 95% CI [1.89, 3.83]	*p* = 0.571
Daily number of cups	1.81(1.87) 95% CI [1.43, 2.57]	1.32 (1.05) 95% CI [0.714, 1.30]	*p* = 0.125	1.38 (1.13) 95% CI [0.678, 1.32]	1.5 (1.65) 95% CI [0.356, 1.64]	*p* = 0.703

**Table 4:** Changes in dietary habits among the 6-year-old children (Group 1 and Group 2) at baseline and at 36 months.

	Group 1 (*n* = 48)	(MI Varnish)	Group 2 (*n* = 48)	(Control)
	At Baseline (%)	At 36 Months (%)	At Baseline (%)	At 36 Months (%)
Snacking (*p*-value)	6.25 (*n* = 3)—Not snacking 4.17 (*n* = 2)—Only fruits 89.58 (*n* = 43)—Sugary snacks	8.33 (*n* = 4)—Not snacking 16.67 (*n* = 8)—Only fruits 75 (*n* = 36)—Sugary snacks (*p* = 0.106)	6.25 (*n* = 3)—Not snacking 10.42 (*n* = 5)—Only fruits 81.25 (*n* = 39)—Sugary snacks	2.08 (*n* = 1)—Not snacking 12.5 (*n* = 6)—Only fruits 85.42 (*n* = 41)—Sugary snacks (*p* = 0.275)
Consumption of soft drinks (*p*-value)	60.41 (*n* = 29)—Not consuming 39.59 (*n* = 19)—Consuming	62.5 (*n* = 30)—Not consuming 37.5 (*n* = 18)— Consuming (*p* = 0.842)	50 (*n* = 24)—Not consuming 50 (*n* = 24)—Consuming	37.5 (*n* = 18)—Not consuming 62.5 (*n* = 30)—Consuming (*p* = 0.442)
Consumption of tea (*p*-value)	35.41 (*n* = 17)—Not consuming 10.42 (*n* = 5)— Without sugar 54.17 (*n* = 26)— With sugar	33.33 (*n* = 16)—Not consuming 14.58 (*n* = 7)— Without sugar 52.08 (*n* = 25)— With sugar (*p* = 0.706)	22.92 (*n* = 11)—Not consuming 18.75 (*n* = 9)— Without sugar 58.33 (*n* = 28)— With sugar	22.92 (*n* = 11)—Not consuming 22.92 (*n* = 11)— Without sugar 52.02 (*n* = 25)— With sugar (*p* = 0.439)
Daily frequency of snacking, soft drinks, and tea with sugar (“Sugar frequency”)	29.7 (*n* = 14)— 0–2 times daily 45.83 (*n* = 22)—3–5 times daily 25 (*n* = 12)—>5 times daily	43.75 (*n* = 21)— 0–2 times daily 41.67 (*n* = 20)—3–5 times daily 16.67 (*n* = 8)—>5 times daily	27.08 (*n* = 13)— 0–2 times daily 43.75 (*n* = 21)—3–5 times daily 27.08 (*n* = 13)—>5 times daily	27.08 (*n* = 13)—0–2 times daily 45.83 (*n* = 22)—3–5 times daily 25 (*n* = 12)—>5 times daily

**Table 5:** Changes in dietary habits among the 12-year-old children (Group 3 and Group 4) at baseline and at 36 months.

	Group 3 (*n* = 47) (MI Varnish)		Group 4 (*n* = 37) (Control)	
	At Baseline (%)	At 36 Months (%)	At Baseline (%)	At 36 Months (%)
Snacking (*p*-value)	4.3 (*n* = 2)—Not snacking 8.51 (*n* = 4)—Only fruits 87.23 (*n* = 41)—Sugary snacks	4.3 (*n* = 2)—Not snacking 23.4 (*n* = 11)—Only fruits 72.34 (*n* = 34)—Sugary snacks (*p* = 0.066)	2.7 (*n* = 1)—Not snacking 16.22 (*n* = 6)—Only fruits 81.08 (*n* = 30)—Sugary snacks	16.22 (*n* = 6)—Not snacking 16.22 (*n* = 6)—Only fruits 67.57 (*n* = 25)—Sugary snacks (*p* = 0.223)
Consumption of soft drinks (*p*-value)	44.68 (*n* = 21)—Not consuming 53.22 (*n* = 26)—Consuming	27.66 (*n* = 13)—Not consuming 72.34 (*n* = 34)—Consuming (*p* = 0.572)	37.84 (*n* = 14)—Not consuming 62.16 (*n* = 23)—Consuming	35.14 (*n* = 13)—Not consuming 64.86 (*n* = 24)—Consuming (*p* = 0.117)
Consumption of tea (*p*-value)	8.51 (*n* = 4)—Not consuming 14.89 (*n* = 7)— Without sugar 76.6 (*n* = 36)— With sugar	14.89 (*n* = 7)—Not consuming 14.89 (*n* = 7)— Without sugar 70.21 (*n* = 33)— With sugar (*p* = 0.317)	16.25 (*n* = 6)—Not consuming 16.25 (*n* = 6)— Without sugar 67.57 (*n* = 25)— With sugar	16.25 (*n* = 6)—Not consuming 24.32 (*n* = 9)— Without sugar 59.46 (*n* = 22)— With sugar (*p* = 0.527)
Daily frequency of snacking, soft drinks, and tea with sugar (“Sugar frequency”)	23.4 (*n* = 11)—0–2 times daily 34.04 (*n* = 16)—3–5 times daily 42.56 (*n* = 20)—>5 times daily	10.63 (*n* = 5)—0–2 times daily 59.57 (*n* = 28)—3–5 times daily 30 (*n* = 14)—>5 times daily	24.32 (*n* = 9)—0–2 times daily 48.65 (*n* = 18)—3–5 times daily 27.02 (*n* = 10)—>5 times daily	34.43 (*n* = 12)—0–2 times daily 37.84 (*n* = 14)—3–5 times daily 29.73 (*n* = 11)—>5 times daily

**Table 6:** The correlation analysis of sugar influence on caries development in 6-year-olds (Group 1 and Group 2) and 12-years-olds (Group 3 and Group 4).

	Group 1 (*n* = 48) MI Varnish		Group 2 (*n* = 48) Control		Group 3 (*n* = 47) MI Varnish		Group 4 (*n* = 37) Control	
	At Baseline	36 Months	At Baseline	36 Months	at Baseline	36 Months	At Baseline	36 Months
Caries1 and “Sugar frequency”	*r* = 0.321 *p* = 0.026	*r* = 0.120 *p* = 0.418	*r* = −0.021 *p* = 0.886	*r* = 0.09 *p* = 0.529	*r* = −0.030 *p* = 0.843	*r* = −0.139 *p* = 0.352	*r* = 0.303 *p* = 0.069	*r* = 0.307 *p* = 0.065
Caries1 and “Sugar daily amount”	*r* = 0.121 *p* = 0.475	*r* = 0.243 *p* = 0.147	*r* = 0.099 *p* = 0.513	*r* = −0.130 *p* = 0.391	*r* = −0.101 *p* = 0.510	*r* = −0.076 *p* = 0.620	*r* = 0.022 *p* = 0.903	*r* = 0.122 *p* = 0.499
Caries2 and “Sugar frequency”	*r* = 0.262 *p* = 0.072	*r* = 0.086 *p* = 0.561	*r* = −0.092 *p* = 0.532	*r* = 0.030 *p* = 0.841	*r* = −0.059 *p* = 0.695	*r* = −0.004 *p* = 0.981	*r* = 0.303 *p* = 0.069	*r* = 0.307 *p* = 0.065
Caries2 and “Sugar daily amount”	*r* = 0.177 *p* = 0.293	*r* = 0.256 *p* = 0.126	*r* = 0.034 *p* = 0.822	*r* = −0.132 *p* = 0.381	*r* = −0.050 *p* = 0.743	*r* = −0.015 *p* = 0.924	*r* = 0.022 *p* = 0.903	*r* = 0.122 *p* = 0.499

## Data Availability

The dataset generated during and analyzed during the current study are available upon request from Jekaterina Gudkina (j.gudkina@inbox.lv).

## References

[1] Cappelli D.P., Mobley C.C. (2017). Association between Sugar Intake, Oral Health, and the Impact on Overall Health: Raising Public Awareness. Curr. Oral. Health. Rep..

[2] Gudkina J., Brinkmane A., Abrams S.H., Amaechi B.T. (2016). Factors influencing the caries experience of 6 and 12 year old children in Riga Latvia. Stomatologija.

[3] Maldupa I., Sopule A., Uribe S.E., Brinkmane A., Senakola E. (2021). Caries prevalence and severity for 12-year-old children in Latvia. Int. Dent. J..

[4] Gudkina J., Amaechi B.T., Abrams S.H., Brinkmane A., Jelisejeva I. (2019). Caries Increment and Oral Hygiene Changes in 6- and 12-Year-Old Children in Riga, Latvia: A 3-Year Follow-Up Report Using ICDAS II and RADKE Criteria. Eur. J. Dent..

[5] Alkilzy M., Santamaria R.M., Schmoeckel J., Splieth C.H. (2018). Treatment of Carious Lesions Using Self-Assembling Peptides. Adv. Dent. Res..

[6] Santos de Oliveira de Sousa F., Pires dos Santos A.P., Nadanovsky P., Hujoel P., Cunha-Cruz J., Heloisa de Oliveira B. (2019). Fluoride Varnish and Dental Caries in Preschoolers: A Systematic Review and Meta-Analysis. Caries. Res..

[7] González-Cabezas C., Fernández C.E. (2018). Recent Advances in Remineralization Therapies for Caries Lesions. Adv. Dent. Res..

[8] Oliveira G.M.S., Ritter A.V., Heymann H.O., Swift E., Donovan T., Brock G., Wright T. (2014). Remineralization Effect of CPP-ACP and Fluoride for White Spot Lesions in vitro. J. Dent..

[9] Malone H.E., Nicholl H., Coyne I. (2016). Fundamentals of estimating sample size. Nurse Res..

[10] Charan J., Biswas T. (2013). How to Calculate Sample Size for Different Study Designs in Medical Research?. Indian J. Psychol. Med..

[11] Cohen J. (1960). A coefficient of agreement of nominal scales. Psychol. Bull..

[12] Jablonski-Momeni A., Stachniss V., Ricketts D.N., Heinzel-Gutenbrunner M., Pieper K. (2008). Reproducibility and accuracy of the ICDAS-II for detection of occlusal caries in vitro. Caries. Res..

[13] WHO (1997). Oral Health Surveys: Basic Methods.

[14] Briefel R.R., Johnson C.L. (2004). Secular Trends in Dietary Intake in the United State.Annu. Rev. Nutr..

[15] Adair L.S., Popkin B.M. (2005). Are child eating patterns being transformed globally?. Obes. Res..

[16] Johansson I., Lif Holgerson P., Kressin N.R., Nunn M.E., Tanner A.C. (2010). Snacking Habits and Caries in Young Children. Caries. Res..

[17] Moynihan P. (2016). Sugars and Dental Caries: Evidence for Setting a Recommended Threshold for Intake. Adv. Nutr..

[18] Moynihan P.J., Kelly S.A.M. (2014). Effect on Caries of Restricting Sugars Intake: Systematic Review to Inform WHO Guidelines. J. Dent. Res..

[19] Amaechi B.T. (2017). Remineralization- the buzzword for early MI caries management. Br. Dent. J..

[20] Marinho V.C., Worthington H.V., Walsh T., Clarkson J.E. (2013). Fluoride varnishes for preventing dental caries in children and adolescents. Cochrane Database Syst. Rev..

[21] Baik A., Alamoudi N., El-Housseiny A., Altuwirqi A. (2021). Fluoride varnishes for preventing occlusal dental caries: A review. Dent. J..

[22] Yu O.Y., Lam W.Y.-H., Duangthip D., Chu C.-H. (2021). Nonrestorative management of dental caries. Dent. J..

[23] Huang G.J., Roloff-Chiang B., Mills B.E., Shalchi S., Spiekerman C., Korpak A.M., Starrett J.L., Greenlee G.M., Drangsholt R.J., Matunas J.C. (2013). Effectiveness of MI Paste Plus and PreviDent fluoride varnish for treatment of white spot lesions: A randomized controlled trial. Am. J. Orthod. Dentofac. Orthop..

[24] Swietlicka I., Kuc D., Swietlicki M., Arczewska M., Muszyński S., Tomaszewska E., Prószyński A., Gołacki K., Błaszczak J., Cieślak K. (2020). Near-Surface Studies of the Changes to the Structure and Mechanical Properties of Human Enamel under the Action of Fluoride Varnish Containing CPP–ACP Compound. Biomolecules.

[25] Zeitouny M., Fayyad-Kazan H., Tassery H., Fayyad-Kazan H. (2020). In Vitro Influence of Prophylaxis Cleaning on Enamel Remineralization with Casein Phosphopeptide-Amorphous Calcium Phosphate. J. Oral. Maxillofac. Res..

[26] Varma V., Hegde S.K., Bhat S.S., Sargod S.S., Ajay Rao H.T. (2019). Comparative Evaluation of Remineralization Potential of Two Varnishes Containing CPP–ACP and Tricalcium Phosphate: An In Vitro Study. Int. J. Clin. Pediatr. Dent..

[27] Kooshki F., Pajoohan S., Kamareh S. (2019). Effects of treatment with three types of varnish remineralizing agents on the microhardness of demineralized enamel surface. Clin. Exp. Dent..

[28] Dumbryte I., Linkeviciene L., Linkevicius T., Malinauskas M. (2017). Does orthodontic debonding lead to tooth sensitivity? Comparison of teeth with and without visible enamel microcracks. Am. J. Orthod. Dentofac. Orthop..

[29] Scribante A., Gallo S., Celmare R.L., D’Antò V., Grippaudo C., Gandini P., Sfondrini M.S. (2020). Orthodontic debonding and tooth sensitivity of anterior and posterior teeth. Angle Orthod..

